# Supplementary material for: Extending the Shelf-Life of Immunoassay-Based Microfluidic Chips through Freeze-Drying Sublimation Techniques
Source: Sensors (Basel). 2023 Oct 17;23(20):8524. doi: 10.3390/s23208524 (PMC10610996; doi:10.3390/s23208524)
Supplement: Supplementary file 1 [file sensors-23-08524-s001.zip › sensors-2655639-supplementary.pdf]

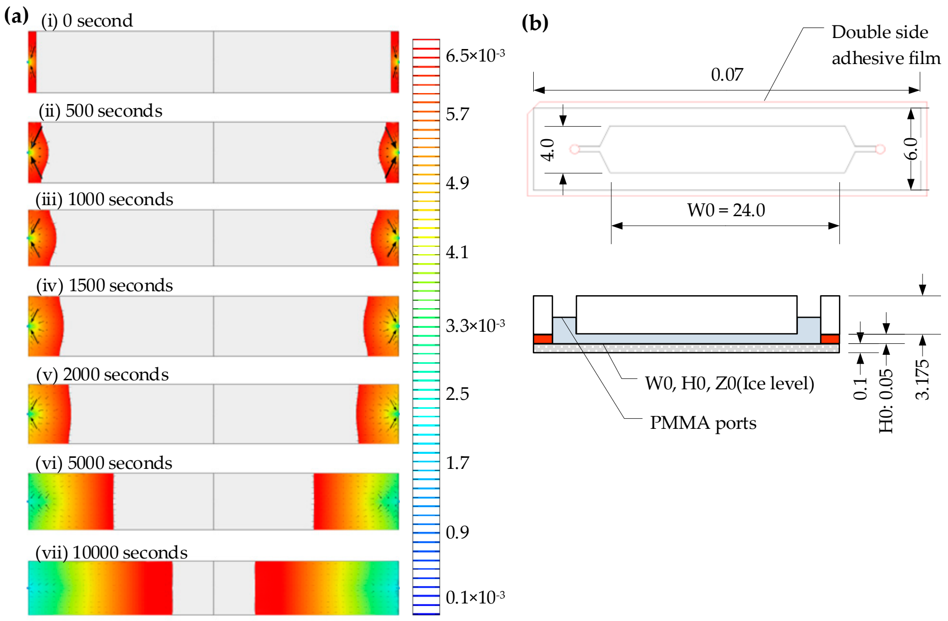

(c)

	Parameter	Abbreviation	Unit	Ref. value
1	Ice density	rho_ice	kg/m^3	920
2	Ice thermal conductivity	k_ice	W/(m×K)	2.56
3	Ice heat capacity	Cp_ice	kJ/(kg×K)	1.8
4	Latent heat of sublimation	L_ice	MJ/kg	2.84
5	Effective diffusivity of vapor	D_vap	m^2/s	0.05
6	Water molar mass	M_water	g/mol	18
7	Air molar mass	M_air	g/mol	29
8	Initial ice temperature	T_ice	K	230
9	Condenser temperature	T_c	K	200
10	Wall heat transfer coefficient	h_wall	W/(m^2×K)	10
11	External temperature	T0	K	230
12	Vacuum chamber pressure	P0	Pa	20
eq1	Water partial pressure	p_water	-	c×R_const×T (R_const is gas constant)
eq2	Absolute pressure	p	-	P0+p_water
eq3	Vapor molar mass	M_vap	-	(P0×M_air+p_water×M_water)/p
eq4	Initial vapor concentration at ice/vapor interface	c_ice	-	p_eq(T_ice)/(R_const×T_ice)
eq5	Vapor concentration at condenser	c_c	-	p_eq(T_c)/(R_const×T_c)
eq6	Initial vapor concentration	c_init	-	c_ice+(c_c-c_ice) ×[(Zg-Z0)/(H0-Z0)]$_{ice}$
eq7	Initial temperature distribution	T_init	-	T_ice+(T_c-T_ice) ×[(Zg-Z0)/(H0-Z0)]$_{ice}$
eq8	Heat flux at chip bottom	Q_in	-	if(x>0,5.5[kW/m^2] ×((x/W0)^2+(y/W0)^2),0)
eq9	Sublimation interface velocity	V_s	-	[T_lm]$_{sublimation}$/(rho_ice×L_ice)

Figure S1. (a) simulation outcomes over a time span ranging from 0 to 10,000 seconds. (b)chip dimension for simulation. (c) 12 parameters and 9 equations are used to simulate frozen dry of the chip.

Table S1. Surface chemistry procedure for microfluidic device at the surface of the channel.

	Step	Methodology	Reaction Time and Temperature Condition
**1**	Plasma Treatment	Glass, Air plasma	60 seconds
**2**	Salinization	Ethanol: 3 MPS = 5ml : 200 μl (Dipping)	60 minutes at room temperature
**3**	Rinse and Soaking	Rinse with ethanol, and backing at 120 °C Ethanol washing after assembling the channel.	60 minutes
**4**	GMBS	Ethanol washing before using Ethanol : GMBS = 5 ml : 14 μl	60 minutes at room temperature
**5**	Rinse (I)	Rinse with Ethanol	-
**6**	Rinse (II)	Wipe the device and change petri dishes Rinse with PBS	-
**7**	NeutraAvidin	NeutraAvidin : PBS = 1 : 100 (50 μl:5 ml)	At 4°C for at least 60 minutes
**8**	Passivation with BSA	Rinse with PBS Passivation at PBS/BSA (1 %) Rinse with PBS	-
**9**	Anti CD4	Anti CD4 - biotinlayted at PBS = 1:9 (5 μl : 450 μl) After 1st injection: 30 min at 4°C After 2nd injection: 30 min at room temperature	-
**10**	Rinse	Rinse with PBS	-
